# High incidence of cleft palate and vomer deformities in patients with Eustachian tube dysfunction

**DOI:** 10.1038/s41598-022-14011-5

**Published:** 2022-06-16

**Authors:** Seong Hoon Bae, Jun-Young Kim, Mincheol Jeong, In Seok Moon, Sung Huhn Kim, Jae Young Choi, Jinsei Jung

**Affiliations:** 1 Department of Otorhinolaryngology, Yonsei University College of Medicine, Severance Hospital, Yonsei University Health System, 50 Yonsei-ro, Seodaemun-gu, Seoul, 120-752 Republic of Korea; 2 Department of Oral & Maxillofacial Surgery, College of Dentistry, Yonsei University, Seoul, Korea

**Keywords:** Anatomy, Diseases, Risk factors, Signs and symptoms

## Abstract

Although the cleft palate is regarded as a contraindication for Eustachian tube ballooning, the presence of submucosal cleft palate may be overlooked while diagnosing Eustachian tube dysfunction. Therefore, we aimed to determine the incidence of the presence of a hard palate bony notch and vomer defect, which indicate the presence of submucosal cleft palate in patients with Eustachian tube dysfunction. In the Eustachian tube dysfunction group (n = 28), 4 patients (14.3%) exhibited a hard palate bony notch and a concurrent vomer defect. Three of them exhibited the presence of occult submucosal cleft palate, which had not been diagnosed previously. None of the control group (n = 39) showed any of these findings. The hard palate length of patients in the Eustachian tube dysfunction group was significantly lesser than that of those in the control group (34.2 ± 5.6 mm vs. 37.2 ± 2.1 mm, *P* = 0.016). Patients with Eustachian tube dysfunction have a high incidence of submucosal cleft palate and its occult variant, which are challenging to diagnose without any preexisting suspicion. Clinicians should evaluate the hard palate and vomer to exclude the presence of occult submucosal cleft palate while diagnosing Eustachian tube dysfunction.

## Introduction

The correlation between cleft palate and Eustachian tube dysfunction (ETD) was revealed several decades ago^1,2^. ETD was found in 79% of children and 31% of adults who were diagnosed with cleft palate regardless of their type^3,4^. Clefts of the lip and palate can be categorized as cleft lip with or without cleft palate and isolated cleft palate^5^. The diagnosis of cleft palate is not challenging because of its obvious features. In addition, the typical symptoms, such as problems in speech and swallowing, also provide clues in diagnosis while screening newborns. However, some patients with submucosal cleft palate (SMCP) are diagnosed late because the cleft is hidden beneath the normal mucosal surface, and therefore, the symptoms are often subclinical. Furthermore, some patients with SMCP who do not meet the triad of clinical criteria (presence of uvula bifida, absence of posterior nasal spine in the hard palate, and presence of zona pellucida in the soft palate) by Calnan exhibit the so-called “occult” SMCP^6–8^. Although the degrees of deformity in the cleft palate differ, all of them are reported to be related to ETD and otitis media^3,4,7^.

ETD was recently revisited after the introduction of Eustachian tube balloon dilatation (EBD) surgery. According to Plaza et al., cleft lip/palate is an absolute contraindication for EBD because ETD in the cleft palate is related to the dysfunction of the tensor veli palatini rather than the mechanical obstruction of the tubal lumen^9,10^. However, the diagnosis of SMCP is challenging if it is not being considered in the differential diagnosis. Furthermore, computed tomography (CT), which is not recommended before EBD according to the clinical consensus statement of 2019, is often essential to diagnose hard plate deformities^11^.

Nevertheless, the incidence of SMCP in patients with obstructive ETD has not been reported. Therefore, this study aimed to identify the prevalence of hard palate or vomer deformity in patients with ETD because the presence of the hard palate notch (HP notch) indicates the incomplete fusion of tensor veli and levator veli palatini muscles that significantly affect Eustachian tube dynamics^12^. In addition, vomer malformation is reported in approximately 92% of the patients with SMCP^13^. Therefore, we also assessed the incidence of possible SMCP (including occult SMCP) in patients with ETD. The results of this study could make the clinicians aware of the possibility of SMCP while diagnosing ETD.

## Results

### High incidence of the HP notch in the ETD group

**Figure 1:**
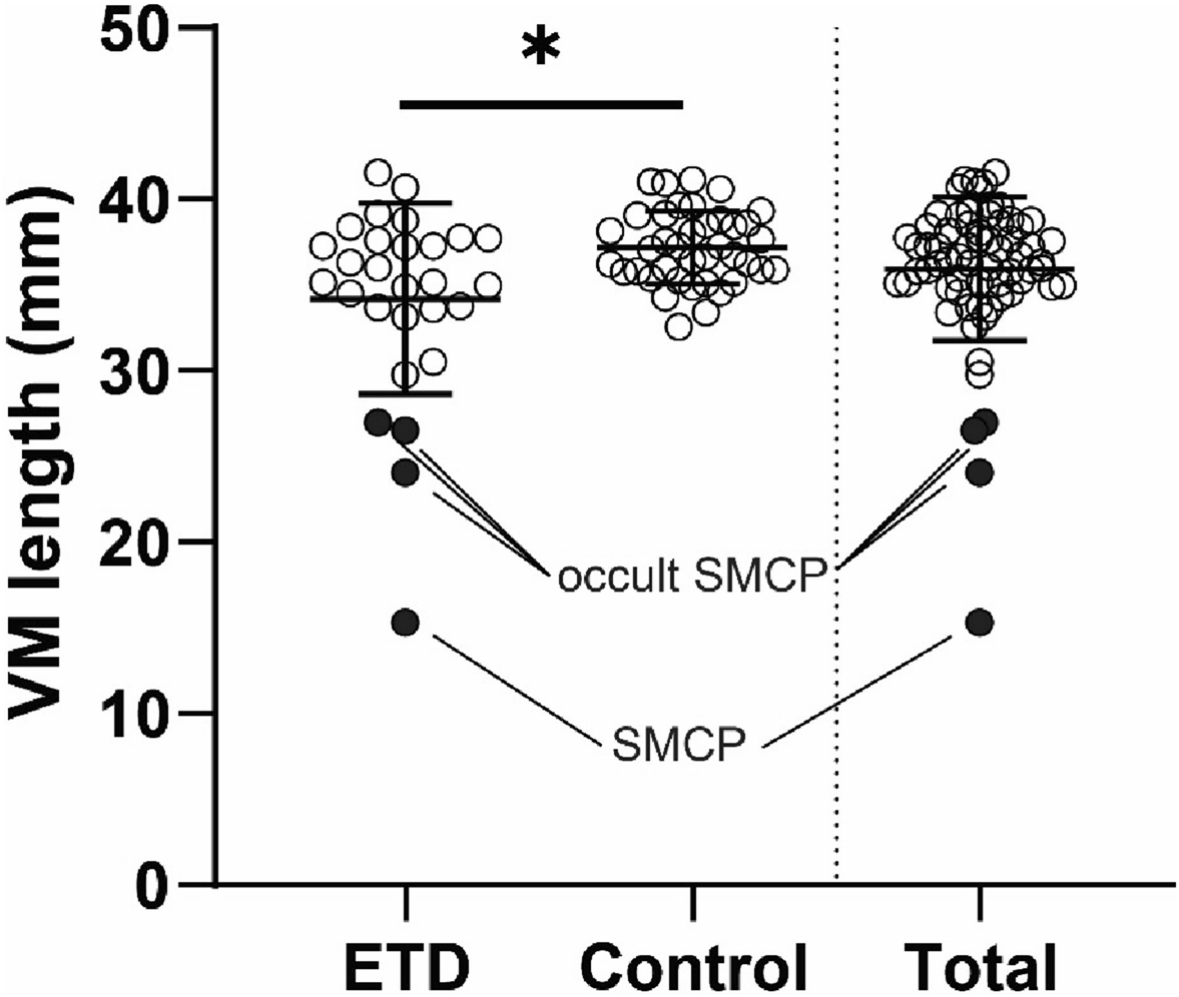
Patients in the ETD group with significantly shorter vomer-maxilla fusion length than those in the control group. Hollow circles indicate the VM length of each subject. Black circles indicate patients with a hard palate bony notch. *VM length* vomer-maxilla fusion length, *ETD* Eustachian tube dysfunction group, *Control* control group, *SMCP* submucosal cleft palate. *P < 0.05.

The demographic factors of age (*P* = 0.560) and sex (*P* = 0.431) were not significantly different between the two groups (Table 1). The mean age of the enrolled patients was 50.8 and 53.5 years in the ETD and control groups, respectively. The proportions of men were 39.3% and 28.2% in the ETD and control groups, respectively. In the ETD group, 60.7% of patients had a history of recurrent otitis media, 89.3% had middle ear pressure < − 50 daPa, and 92.9% had pars tensa retraction. Among the 28 patients with ETD, 14.3% (n = 4) had a V-shaped HP notch, and all of them concomitantly exhibited vomer deformation. In contrast, no patient in the control group had the V-shaped HP notch or vomer deformation. The incidence of HP notch was significantly different between the ETD and control groups (*P* = 0.027). Furthermore, the vomer-maxilla fusion length (VM length) of patients in the ETD group was significantly lesser (34.2 ± 5.6 mm) than that of patients in the control group (37.2 ± 2.1 mm; *P* = 0.011; Fig. 1). The average VM length of the 67 enrolled patients (28 in the ETD group and 39 in the control group) was 35.9 ± 4.2 mm. Two (33.3%) and four (66.7%) patients in the ETD and control groups, respectively, had greater VM length than 1 SD (> 40.1 mm), which was comparable to the proportion of patients in each group (41.8% and 58.2%, respectively). Conversely, six patients in the ETD group, including four patients with an HP notch, had a VM length lesser than 1 SD (< 31.7 mm). In addition, VM length was not significantly different between the two groups when excluding four patients with HP notch (*P* = 0.118).

**Table 1:** Comparative evaluation of the ETD and control groups.

	ETD group	Control group	*P*-value
Age, mean (SD), years	50.8 (17.4)	53.5 (19.5)	0.560
Men, n (%)	11 (39.3)	11 (28.2)	0.431
**Symptoms, n (%)**			
Recurrent otitis media with effusion	17 (60.7)	0	
Middle ear pressure < − 50 daPa	25 (89.3)	0	
Retracted drum	26 (92.9)	0	
Tinnitus	0	15 (38.5)	Not applicable
External auditory canal chole/mass	0	8 (20.5)	
Otitis externa	0	8 (20.5)	
Other external auditory canal problem	0	8 (20.5)	
Hard palate notch, n (%)	4 (14.3)	0.0	0.027*
Vomer deformity, n (%)	4 (14.3)	0.0	0.027*
Vomer-maxilla fusion length, mean (SD), mm	34.2 (5.6)	37.2 (2.1)	0.016*^a^
Patients, n	28	39	

*ETD* Eustachian tube dysfunction, *SD* standard deviation.

*P-value < 0.05.

^a^Mann–Whitney test.

### Clinical characteristics of patients with an HP notch

**Figure 2:**
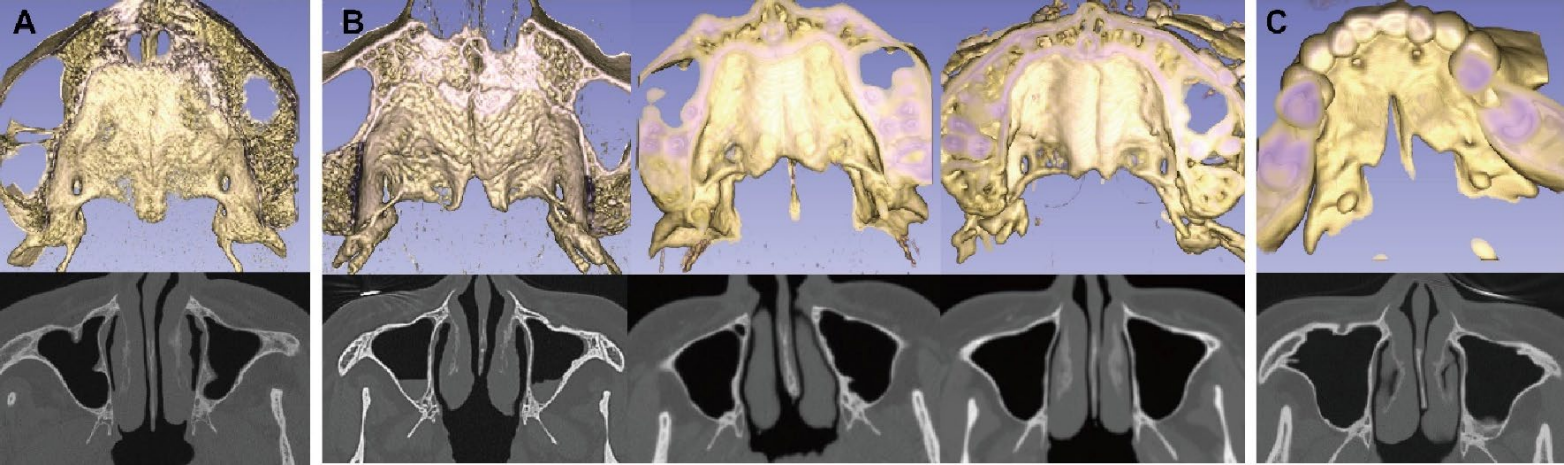
Images of the hard palate bony notch (top) and vomer deformity (bottom). (**A**) Normal hard palate and vomer in patients in the control group. (**B**) Three patients with occult submucosal cleft palate. (**C**) Patient with a history of submucosal cleft palate diagnosis.

**Figure 3:**
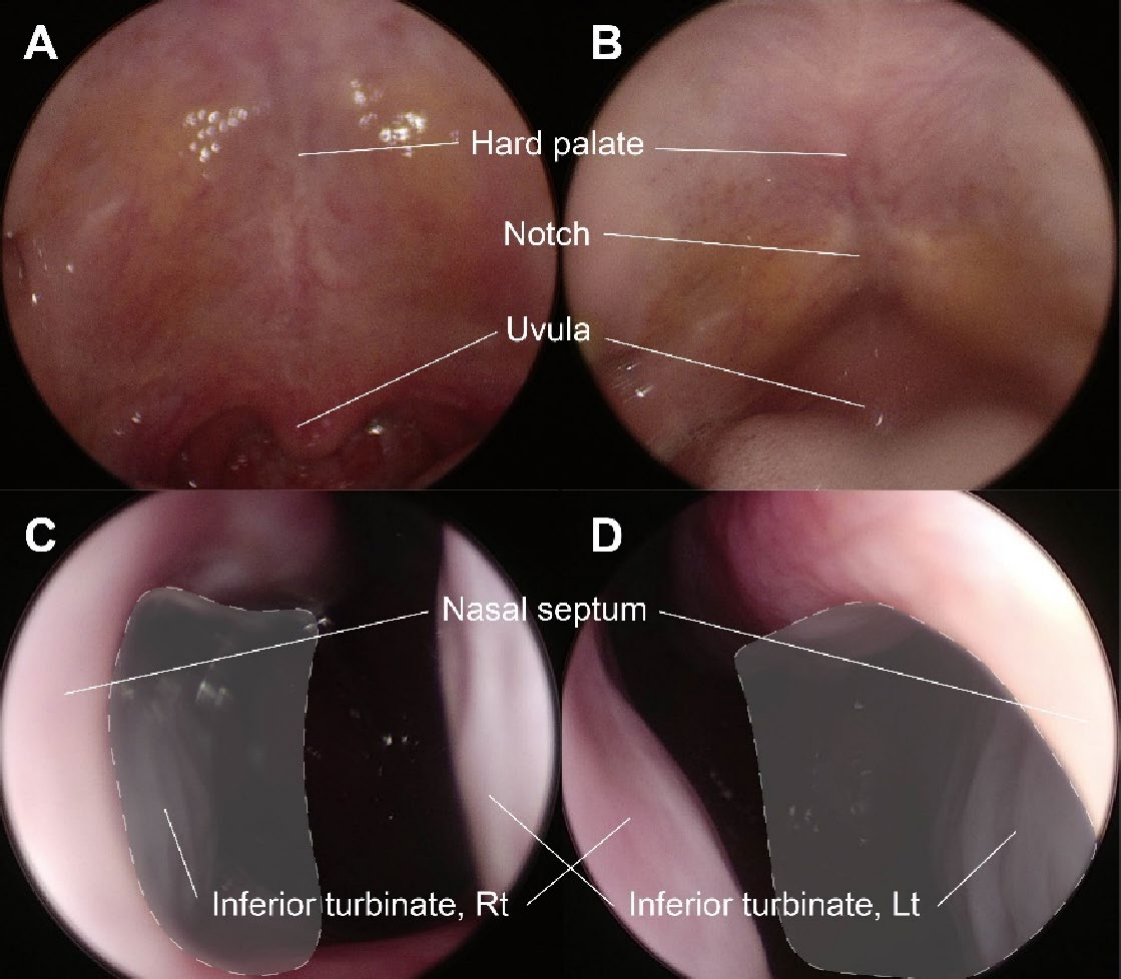
Representative nasal endoscopy images of a patient with occult submucosal cleft palate. (**A**) Normal hard palate (without zona pellucida) and uvula in the resting state and (**B**) during phonation. Vomer deformity in the inferior-posterior nasal septum defect. Bilateral inferior turbinates in a single field. (**C**) Nasal endoscopy of the Lt nostril. (**D**) Nasal endoscopy of the Rt nostril. Gray areas with dotted marginal lines are defective portions of the nasal septum. *Lt* left, *Rt* right.

Of the four patients with an HP notch, only one who had undergone palatoplasty in childhood reported a history of SMCP. The other three patients had no velopharyngeal or speech problems in their lifetime; none of them exhibited the presence of zona pellucida, while one had bifid uvula, which implies that these patients had occult SMCP. Unlike those who were diagnosed with SMCP in childhood, patients with occult SMCP exhibited otological problems in adulthood. In addition, they had a milder HP notch compared to the patient with SMCP (Fig. 2). All patients with an HP notch had a vomer deformity. The inferior-posterior nasal septum was absent, which enabled the visualization of the bilateral inferior turbinates in a single field (Fig. 3). One patient with occult SMCP had unilateral otological problems, whereas the other 2 with occult SMCP and 1 patient with SMCP had bilateral otological problems. All patients with an HP notch exhibited recurrent otitis media and a middle ear pressure below − 50 daPa. These patients also showed a retracted tympanic membrane, except for one patient with occult SMCP (Table 2).

**Table 2:** Detailed characteristics of patients with submucosal cleft palate.

Patient	Age	Sex	Triads	SOM	TM	MEP (daPa)	VM (mm)
SMCP 1	16	F	HP ZP BU	Bil	Bil	R: − 76 L: − 306	15.31
oSMCP 1	21	F	HP	Left	No	R: − 1 L: − 308	26.53
oSMCP 2	24	F	HP BU	Bil	Bil	R: − 18 L: − 142	24.05
oSMCP 3	56	F	HP	Bil	Bil	R: − 61 L: type B	26.98

*Triads* Calnan triads, *SOM* history of recurrent serous otitis media, *TM* tympanic membrane retraction, *MEP* middle ear pressure, *VM* vomer-maxilla fusion length, *SMCP* submucosal cleft palate, *oSMCP* occult submucosal cleft palate, *F* female, *HP* hard palate notch, *ZP* zona pellucida, *BU* bifid uvula, *Bil* bilateral, *R* right, *L* left.

## Discussion

In this study, the HP notch was identified in four patients (14.3%) in the ETD group and none in the control group. Among them, only one patient was diagnosed with SMCP according to the triad of clinical criteria by Calnan. Interestingly, the other three patients with HP notches who had occult SMCP were not diagnosed throughout their lifetime. Although the incidence of occult SMCP has not been reported, the incidence of the presence of the HP notch in the ETD group is reported to be remarkably high considering that the incidence of SMCP is reported to range from 0.02 to 0.08% in newborn screening^14,15^. In addition, Reiter et al. reported the incidence of occult SMCP as 23.9%, who satisfied the triad of clinical criteria by Calnan out of 439 operated patients with SMCP. Interestingly, this incidence is similar to the incidence of SMCP and occult SMCP in patients in the ETD group in our study (SMCP, 25%; occult SMCP, 75%).

Patients with SMCP, including those with occult SMCP who only have hard palate bony defects without the other two clinical manifestations, tend to be diagnosed later, as indicated in previous studies^16–18^. Since up to 55% of patients with SMCP can be asymptomatic, they may not be aware of their deformity, similar to the patients in our study^7,19–21^. Sommerland et al. suggested a grading system for SMCP that considered the presence of the HP notch, bifid uvula, and intraoperative muscular findings. The total score (0–9) for each of the three findings was defined as the SMCP score; they categorized patients with occult SMCP as those with 1–3 SMCP scores^22^. Patients who only had an HP bony notch in our study could also be diagnosed with occult SMCP, according to Sommerland et al. In addition, since we focused only on the hard palate in the CT scan, the SMCP score of such patients may be higher if we thoroughly evaluated them prospectively. However, this remains inconclusive because their soft palate musculature was not assessed. Considering the symptoms of ETD, these patients may have velopharyngeal dysfunction, which may be evident after a meticulous evaluation is performed.

Clinicians may overlook the presence of SMCP, though the cleft palate is a contraindication for EBD^10^. According to the clinical consensus statement for EBD published in 2019, establishing a diagnosis of obstructive ETD requires the exclusion of patulous ETD, temporomandibular joint disorders, extrinsic obstruction of the Eustachian tube, superior semicircular canal dehiscence, and endolymphatic hydrops^11^. Additionally, a thorough recording of history and physical examination are essential to diagnose ETD, though SMCP is not mentioned specifically. Most cases of cleft palate are diagnosed on postnatal physical examination; of these, 7.7% are cases of SMCP, which can be overlooked by the physician^16^. Moreover, the diagnosis of occult SMCP is even more challenging when patients have subclinical velopharyngeal symptoms. Although the cleft palate is a contraindication for EBD, previous studies on EBD may not have considered the possibility of SMCP^10,23–27^. This may be because clinicians are prejudiced that cleft palate is an obvious disease that is less likely to be harbored until adulthood. Importantly, patients with occult SMCP in our study developed recurrent otitis media in adulthood, which can further confound clinicians in suspecting an occult SMCP. Thus, clinicians should be aware of the possibility of SMCP when diagnosing ETD. Given the results of this study, a vomer examination using a nasal endoscope and CT for determining the presence of an HP notch are recommended in routine clinical evaluation.

### Limitations

This study has several limitations. First, we retrospectively reviewed medical charts and CT images. Thorough physical examination, including the evaluation of velopharyngeal function and magnetic resonance imaging, may support our results. Second, only patients with ETD who satisfied the inclusion criteria were enrolled in this study. Since ETD is closely related to several diseases of the middle ear, investigation of the incidence of SMCP and/or occult SMCP should include evaluation for the presence of other middle ear diseases (e.g., tympanic membrane perforation) in future studies^28,29^. In addition, although theoretically obvious, there is still a lack of clinical evidence that EBD may not be effective in patients with cleft palate. A retrospective review of patients who underwent EBD, especially refractory cases, can directly provide evidence to clarify if EBD is contraindicated in patients with cleft palate, including those with SMCP.

### Conclusion

In conclusion, among patients with ETD, 14.3% exhibited an HP notch and a concomitant vomer deformity. However, no patient in the control group exhibited these findings. Given the results of this study, clinicians must be aware of the possibility of occult SMCP while diagnosing ETD, particularly when determining patients indicated for EBD. Further, the presence of the HP notch and vomer examinations should be routinely performed.

### Clinical recommendations

Hard palate and vomer should be evaluated when diagnosing ETD to rule out SMCP. Therefore, a CT scan and nasal endoscopy are recommended at least before surgical intervention.

## Methods

### Patient enrollment

Patients who visited our clinic from September 1, 2021, to January 31, 2022, were retrospectively reviewed. Forty-one patients who met at least two of the following inclusion criteria in one or both ears were considered for enrollment in the ETD group: (1) history of recurrent serous otitis media (more than 2 times the history of ventilation tube insertion in the same ear after 19 years of age), (2) middle ear pressure < 50 daPa, and (3) retraction of pars tensa. Of these, 13 patients with a history of radiation therapy or head and neck cancer were excluded. One patient was excluded because she was diagnosed with eosinophilic otitis media. Consequently, 28 patients were included in the ETD group. Because the temporal bone CT was routinely conducted to diagnose ETD, all included patients had CT scans. Patients who conducted temporal bone CT scans for external auditory canal disease (n = 27) or tinnitus (n = 15) were considered for inclusion in the control group. Of these, three were excluded because their CT scans showed concomitant middle ear diseases, or they had a history of ear surgery performed for chronic otitis media. Ultimately, 39 patients were enrolled in the control group. This study was approved by the Institutional Review Board of Severance Hospital (Seoul, Korea). The requirement for informed consent was waived owing to the retrospective study design (project 4-2022-0084). This study was conducted according to the guidelines of the Declaration of Helsinki.

### Analysis of CT scans

**Figure 4:**
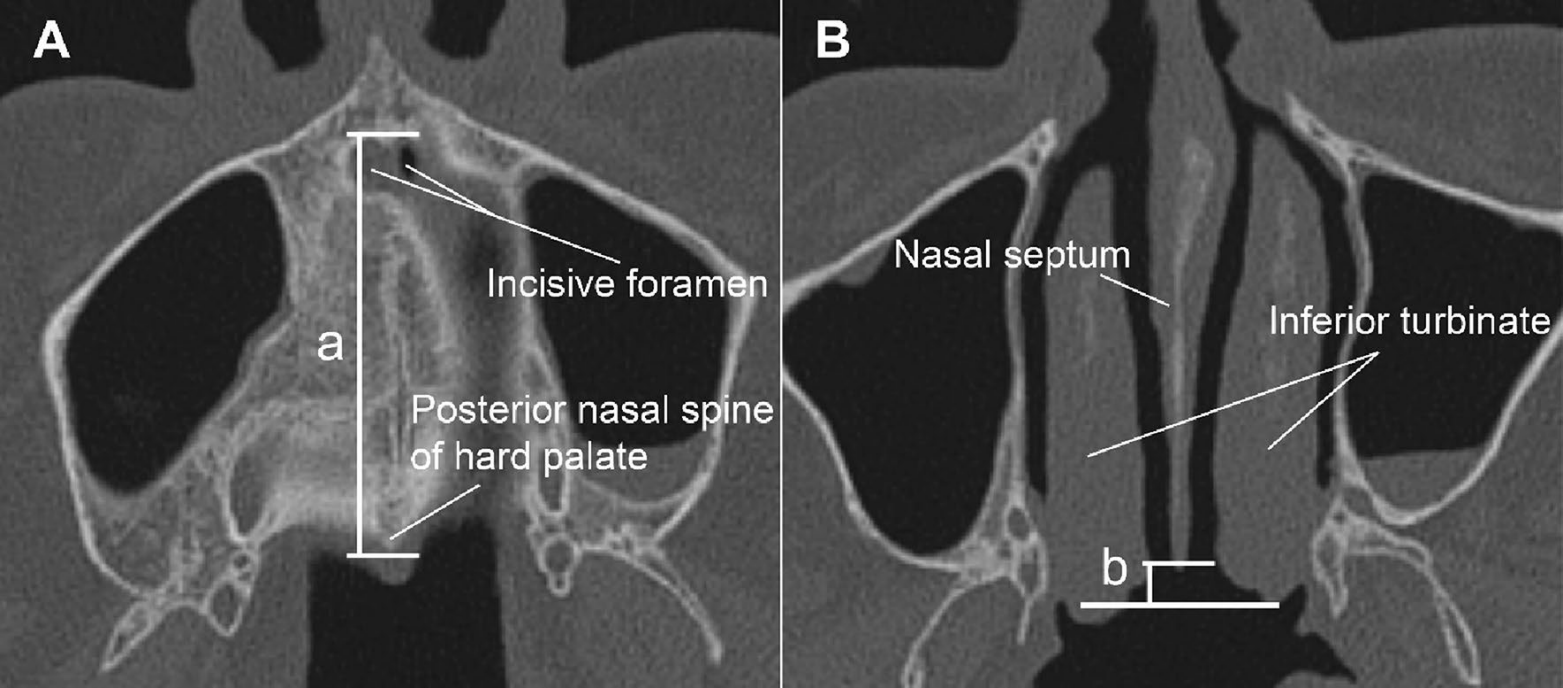
Measurement of vomer-maxilla fusion length (a) and vomer deformity (b) on computed tomography images of patients with a normal hard palate and vomer. (**A**) Vomer-maxilla fusion length is defined as the length from the incisive foramen to the posterior nasal spine of the hard palate. (**B**) Vomer deformity is defined when the posterior edge of the septum is ˃ 5 mm shorter than the shorter side of the posterior end of the inferior turbinate (b > 5 mm).

Bony deformities of the hard palate and vomer were identified using 3D-reconstructed CT images. The presence of the HP notch as a V-shaped defect in the posterior hard palate was evaluated and confirmed using the 3D-slicer software (open-source)^12,13^. Vomer deformity was diagnosed when the posterior edge of the septum was shorter by > 5 mm than the posterior end of the inferior turbinate (shorter side). Hard palate and vomer deformities were quantified by measuring the VM length^13^, which is the distance between the incisive foramen and the posterior nasal spine of the hard palate (Fig. 4). The presence of the HP notch was independently evaluated by two otolaryngologists (S.H.B. and M.J.) and one oral and maxillofacial surgeon (J.Y.K.) and recorded when all the three blinded investigators agreed on its presence.

### Statistical analysis

Fisher’s exact test was used to evaluate the significance of the differences in proportions between the two groups. The Student’s t-test was used to evaluate the difference in continuous variables between the two groups following the Shapiro–Wilk test performed to determine the normality of the data. The Mann–Whitney test was used when the data were not distributed normally. SPSS 25.0 (IBM, Armonk, NY, USA) and Prism 8.0 (GraphPad Software, San Diego, CA, USA) were used for statistical analyses. Data are presented as mean ± SD. Differences with *P*-values less than 0.05 were considered statistically significant.

## Data Availability

The data are available from the corresponding author upon reasonable request.
